# Prevalence and Correlates of Cervical Cancer Prevention Knowledge Among High School Students in Ghana

**DOI:** 10.1177/10901981231217978

**Published:** 2023-12-17

**Authors:** Ama Gyamfua Ampofo, Lisa J Mackenzie, Shadrack Osei Asibey, Christopher Oldmeadow, Allison W Boyes

**Affiliations:** 1 Health Behaviour Research Collaborative, School of Medicine and Public Health, College of Health, Medicine and Wellbeing, The University of Newcastle, Callaghan, New South Wales, Australia; 2 Equity in Health and Wellbeing Research Program, Hunter Medical Research Institute, New Lambton Heights, New South Wales, Australia; 3 Hunter Medical Research Institute, New Lambton Heights, New South Wales, Australia; 4 Faculty of Applied Sciences and Technology, Kumasi Technical University, Kumasi, Ghana

**Keywords:** cervical cancer, prevalence, students, risk factors, correlates, prevention, knowledge

## Abstract

**Introduction:**

Cervical cancer is a preventable yet highly prevalent disease in Africa. Despite female adolescents and young women being a target group for cervical cancer prevention strategies, little research has examined their knowledge of how to prevent the disease. The study aimed to describe: (a) knowledge about cervical cancer prevention and (b) sociodemographic, social, and systemic factors associated with and interacting with knowledge among female senior high school students in Ghana.

**Methods:**

A cross-sectional survey assessed knowledge about (a) risk factors and (b) primary and secondary prevention of cervical cancer among 2,400 female students from 17 public senior high schools in the Ashanti region, Ghana. Descriptive statistics were used to describe knowledge. Linear mixed-effects regression models were used to examine factors associated with knowledge scores.

**Results:**

Knowledge gaps were observed for at least two-thirds (>65%) of students. Most students (mean age = 17) did not know that early sexual debut (before 18 years) is a risk factor for cervical cancer (72%) and that a blood test cannot detect cervical cancer (71%). Students in later stages of senior high school education and those who received sexual health education from teachers and parents had significantly greater cervical cancer knowledge scores than their counterparts. Interactive effects showed that school-based sexual health education was associated with higher knowledge scores than home-based education among students.

**Conclusions:**

Most female senior high school students had gaps in knowledge about cervical cancer prevention. Finding new ways to strengthen the capacity of schools and parents to deliver accurate cervical cancer prevention information is warranted.

## Introduction

Cervical cancer is the fourth most common cancer in women globally and the leading cause of cancer-related death in Africa (Sung et al., 2021). Compared with worldwide estimates, the age-standardized incidence and mortality rate of cervical cancer in Ghana are two times higher (27.4 and 17.8, respectively; Sung et al., 2021). It is also the second most common cancer and the leading cause of cancer deaths among women in Ghana (Sung et al., 2021). The high incidence of cervical cancer could be attributed to increasing exposure to risk factors, including human papillomavirus (HPV) infections (Bruni et al., 2022). Between 2004 and 2018, the percentage of HPV 16/18 infections detected in cervical cancer cases increased from 47% (Awua et al., 2016) to 59% (Bruni et al., 2022) in Ghana. Besides HPV infections, other factors contributing to cervical cancer cases are long-term hormonal contraceptive use, Human Immunodeficiency Virus (HIV), tobacco smoking, multiple sexual partners, multigravida, immunosuppression, and family history of cervical cancer (Bruni et al., 2022).

Despite the growing prevalence of HPV 16/18 infections from 6.2% (Donkoh et al., 2022) to 11.2% (Krings et al., 2019) between 2011 and 2014 among Ghanaian women, the uptake of regular organized cervical screening programs offered by benevolent organizations is less than 12% (Ampofo et al., 2020; Binka et al., 2016). Due to the low coverage of screening services and the absence of symptoms in early-stage cervical cancer, the majority of women with cervical cancer in Ghana have their cancers detected at an advanced stage of the disease (Nartey et al., 2016). Low patronage of cervical screening and late-stage presentation of the disease have been attributed to a number of individual, community and health system barriers, including poor knowledge about the disease (i.e., poor health literacy; Chidyaonga-Maseko et al., 2015; Dunyo et al., 2018). No data exist for HPV vaccination uptake among adolescents and young women, but this is not surprising given that there is no government-mandated HPV vaccination program in Ghana.

Knowledge about cervical cancer prevention is vital for reducing morbidity and mortality associated with the disease. Insufficient knowledge about cervical cancer risk factors, HPV vaccination, and screening is linked to low participation in prevention programs and adverse health outcomes including late diagnosis and treatment (Chidyaonga-Maseko et al., 2015; Ebu et al., 2014; Rugutt, 2014). While previous studies have found inadequate knowledge about cervical cancer risk factors, vaccination, and screening among Ghanaian women, men in the general population, and college students (Binka et al., 2016; Ebu et al., 2014; Gyamfua et al., 2019; Williams & Amoateng, 2012; Zurales et al., 2023), few studies have examined knowledge of the disease among high school students.

There are a disproportionately small number of studies about cervical cancer knowledge among high school girls. Of ten studies identified in the literature (Ayissi et al., 2012; Bardaji et al., 2018; Bowyer et al., 2013; Ifediora & Azuike, 2018; Lee et al., 2014; Mapanga et al., 2019; Martianus et al., 2018; Opoku Agyemang & Manortey, 2018; Poudel & Sumi, 2019; Rashwan et al., 2013), only three were conducted in Western Africa. In Cameroon, more than 70% of girls have been reported to have knowledge about cervical cancer risk factors and HPV vaccination (Ayissi et al., 2012; Bardaji et al., 2018). Conversely, less than 50% of Ghanaian and Nigerian girls have knowledge about cervical cancer risk factors and the Pap test (Ifediora & Azuike, 2018; Opoku Agyemang & Manortey, 2018). While these studies provide some insight, they are limited by the modest sample sizes (<400; Ifediora & Azuike, 2018; Opoku Agyemang & Manortey, 2018) and the use of convenience sampling (Ifediora & Azuike, 2018), which reduces the generalizability and quality of the findings.

Health literacy refers to personal knowledge and competencies that provide the ability to access, comprehend, evaluate, and use the information to improve health and engage in health care services (World Health Organization, 2021). Health literacy is consistently linked with knowledge about sexual and reproductive health (Kilfoyle et al., 2016) and cervical cancer (Kim & Han, 2016) risk behaviors and prevention. According to the adolescent framework for health literacy, three major factors can affect adolescent health literacy: individual characteristics, social environment (i.e., parents and peers), and broader systems (i.e., school and health care; Manganello, 2007). Individual factors crucial to health literacy include age, race, gender, cultural background, and parental education (Fleary et al., 2018). Indeed, previous studies have found that cervical cancer knowledge among high school students may be influenced by individual factors (Li et al., 2009; Mapanga et al., 2019). Evidence suggests that parent-based (Widman et al., 2019), peer-led (Sun et al., 2018), and school-based (Sani et al., 2016) sexual health intervention programs improve adolescent sexual health knowledge of factors such as HIV/acquired immunodeficiency syndrome and contraceptive use. School-based cervical cancer education was found to be effective in improving cervical cancer/HPV infection knowledge among female students in our recent review of English-language publications describing randomized controlled trials (Ampofo et al., 2022). Of the 13 studies included in the review, the majority (*n*=8) were from the United States and assessed knowledge about cervical cancer risk factors and primary prevention (Ampofo et al., 2022).

The World Health Organization’s Health Promoting Schools (HPS) advocacy framework highlights an integrated approach for improving students’ health and well-being (World Health Organization, 1997). The HPS comprises three broad elements: first, integrating health education in school curricula; second, changing the social and physical environment of schools; and third, schools’ engaging with health and education officials, parents, and community (Langford et al., 2016; World Health Organization, 1997). Understanding the current knowledge of high school students and the factors associated with increased knowledge is important for designing and implementing effective school-based cervical cancer education programs, guiding and targeting strategies including the development of educational materials, and identifying channels to reach students (Glanz et al., 2008).

To the authors’ knowledge, no study has comprehensively focused on assessing knowledge about risk factors, primary and secondary prevention of cervical cancer, and how social, family/parental, peer and systemic factors influence knowledge among high school girls across low-, middle-, and high-income settings. Guided by the adolescent health literacy framework and the HPS advocacy framework, this study aimed to describe among female senior high school students: (a) cervical cancer prevention knowledge; (b) individual characteristics, social environment (i.e., parents and peers), and broader systems (i.e., school and health care) associated with cervical cancer prevention knowledge; and (c) how social environment and broader systems interact to affect cervical cancer prevention knowledge.

## Material and Method

### Study Design, Setting, and Period

The University of Newcastle Human Research Ethics Committee reviewed and approved (#H-2020-0378) the protocol and all study materials. A cross-sectional paper-based survey evaluated knowledge about cervical cancer prevention among 2400 high school students from 17 schools in the Ashanti region of Ghana between February and July 2021. This was part of a larger study examining the prevalence of cervical cancer risk factors and HPV vaccination intentions among high school students. Details of the study methods are described elsewhere (Ampofo et al., 2023; see Appendix A).

### Sample Size

A calculated sample of 17 schools with an average of 71 eligible student participants for each school enabled estimation of the proportion of students with sufficient knowledge (defined as a score of 50%, and conservatively assumed to be 50% of the population) with a margin of error of 6%. This calculation assumed an effective sample size of 260 students, resulting from a design effect of 4.55 and an intraclass correlation of 0.05 (Bland, 2015). Allowing for a conservative 50% response rate, an average of 141 students per school was determined and approximated to give a final sample size of 2,400 participants (Bland, 2015).

### Measures

Self-administered study-specific surveys were developed in accordance with the Consensus-based Standards for the selection of health Measurement Instruments (COSMIN) guidelines in three phases (Gagnier et al., 2021).

#### Phase 1: Item Generation and Content Validity

A pool of 23 candidate items drawn from the previous literature and survey instruments assessing cervical cancer and HPV knowledge among adolescents, both in and out of school, were considered for selection (Ayissi et al., 2012; Bardaji et al., 2018; Bowyer et al., 2013; Martianus et al., 2018; Poudel & Sumi, 2019). In line with the recommendations of Lynn (1986), 12 experts including health behavior scientists (*n*=4), epidemiologists (*n*=2), nurses (*n*=2), doctors (*n*=2), and psychologists (n=2) were purposively selected from the research team’s research and clinical networks. A preliminary list of items was provided by email to the experts who (a) indicated the relevance and comprehensibility of each item on a dichotomous scale of “relevant or not relevant” and “comprehensive or not comprehensive” and (b) suggested any additional items or item modification. Based on the experts’ feedback, new items were added, redundant or duplicate items were deleted, complex items were simplified, and response options were revised. Consensus among experts was required before an item was removed. A summary of feedback from the first round of expert review and the revised list of items were provided to the experts, and the process of item review was repeated. This iterative process of expert review resulted in 11 new items being added (e.g., *“Infection with a virus”* was added as a response option to *“Which of the following can lead to cervical cancer?”*), 10 items were deleted (e.g., “*Infection with candidiasis”* was removed as a response option to *“Which of the following can lead to cervical cancer?*”), and 9 items were re-worded (e.g., *“Having lots of abortions”* became *“Having more than one abortion”*). The resultant 34-item preliminary survey was piloted among students.

#### Phase 2: Pilot-Testing and Instrument Refinement

One hundred students from a public senior high school in the Ashanti Region were invited to complete the preliminary survey to assess the feasibility of survey administration (completion rate ≥50%, response burden <20 minutes) and data entry. Following completion of the survey, 20 participants were purposively sampled and invited to participate in a qualitative semi-structured telephone interview conducted by one author (AGA). Interviews were recorded with participants’ consent. Interview questions (see Supplemental File 1) elicited general feedback about the survey, as well as knowledge, beliefs, and attitudes regarding cervical cancer, HPV vaccination, and cervical cancer screening, guided by the Integrated Behavior Model (Montaño & Kasprzyk, 2015). Data saturation was achieved after eight interviews, and no further interviews were conducted. Manual codes were identified and developed into themes by one author (AGA) using the Integrated Behavior Model. Based on findings from the pilot testing, two new items were added (*“Poor personal hygiene”* and *“Contact with blood of a person with cervical cancer”* were added as response options to *“Which of the following can lead to cervical cancer?”)*, existing items were modified (e.g., *“Getting an injection for cervical cancer”* became *“Getting vaccinated with HPV vaccines to prevent cervical cancer*” as a response option to *“Which of the following can reduce a woman’s chance of getting cervical cancer?”*), and re-ordered. The survey was then reformatted.

#### Phase 3: Psychometric Evaluation

The psychometric performance of the knowledge scale was evaluated against the following criteria: (a) internal consistency, i.e., acceptable reliability coefficient (Cronbach’s α > 0.7) and acceptable inter-item and item-total correlations (0.2–0.8) and (b) item analysis (i.e., item-by-item frequency). Reliability was acceptable (Cronbach α = 0.82), and inter-item and item-total correlations indicated that item efficiency was good (*r* = 0.2–0.7). Item-by-item frequency distribution showed a good spread (fairly equal distribution or flat normal distribution) of response options (Streiner et al., 2015). Items below the recommended threshold were assessed for cultural and clinical relevance before deleting them.

This process led to the revised 26-item survey. Components of the final measure are described below and attached as Supplemental File 2.

##### Cervical Cancer Prevention Knowledge

Cervical cancer prevention knowledge was measured by 26 items across 4 elements. Items consisted of evidence-based and non-evidence-based statements about (a) cervical cancer risk factors (12 items; response options were “Yes/No”), (b) primary prevention strategies (5 items; response options were “True/False”), (c) secondary prevention strategies (5 items; response options were “True/False”); and (d) HPV-related knowledge (4 items; response options were “True/False”). Participants’ answers were scored as “1” for a correct response and “0” for an incorrect or incomplete response to statements. An overall knowledge score was calculated as the sum of all responses, and expressed as a score out of 26, with higher scores indicating more knowledge.

##### Participant Characteristics

Seven items measured age, grade (SHS 1–3), ethnicity (Akan/Northerner/Ewe and Guan/Ga-Adangbe/Other), program of study (General Arts/Science/Home Economics/Business/Agriculture/Visual Arts), enrollment status (Boarder/Day), mother/female guardian education (No formal education/Primary education/Secondary and Tertiary education) and father/male guardian education (No formal education/Primary education/Secondary and Tertiary education).

##### Family and Social Factors

Two items measured whether participants had ever received sexual health education from their parents/guardians and friends. Response options were “Yes/No.”

##### Systemic Factors

School system characteristics were assessed by three items: whether participants had ever received sexual health education from their teachers (response options were “Yes/No”); whether their schools organize sexual health education classes (response options were “Yes/No”); and the schools’ locations (i.e., District or Metropolitan/Municipal). Health system influence was assessed by one item that asked whether participants had ever received sexual health education from a health care professional (response options were “Yes/No”).

### Data Analysis

Knowledge was analyzed using descriptive statistics including percentages, frequencies, means, and standard deviation (*SD*). Mean (*SD*) knowledge scores were calculated from the percentage of correct responses. For the purpose of the analysis, the following variables were re-categorized: Ethnicity (Akans vs. Northerner/Ewe/Guans/Ga—Adangbe/Other); and program of study, Science/Home Economics/Agric (STEM—Science, Technology, Engineering and Math,) vs, General Arts/Visual Arts/Business, Non-STEM)).

Linear mixed-effect regression modeling (LMM) was used to analyze the associations between correlates (i.e., demographic characteristics, social factors, and systemic factors) and overall knowledge scores. The LMM included fixed effects for all correlates and a random school-level intercept to model the clustering within schools from the Ashanti Region. Interaction effects between sexual health education received from parents, peers, teachers, school, and health care professionals were included in the LMM to assess whether there was a synergistic effect between any of these prespecified variables. Pairwise interaction terms were formed by multiplying any two variables, with each entered one at a time into the initial model. Interaction terms that improved the model and/or were significant were retained. The formal test of interaction was assessed using the Wald test. Model assumptions were assessed by inspecting residual plots. The confidence interval and significant *p* value were set at 95% and less than 0.05, respectively. The intra-cluster correlation coefficient (ICC) was calculated and presented as an estimate of the between-school variance divided by the sum of the within-school and between-school variance. For this study, the ICC reflects how similar knowledge scores are for students in the same school. If the ICC is close to zero, it is indicative of homogeneity between students in each school and small to no differences in knowledge scores between the samples across all included schools.

## Results

Two thousand four hundred students participated in the study. All 17 schools that were invited agreed to participate, and the target quota of student participants of at least 141 was achieved in each school. Table 1 presents the characteristics of student participants. The average age of participants was 17 years. The most prevalent grade level of participants was SHS level one (46%), and 77% belonged to the Akan tribe. More than half (52%) of participants were General Arts students. Over 60% of participants’ parents had a secondary or tertiary education.

**Table 1.** Characteristics, Social and Systemic Factors of Participants, *N*=2,400.

Characteristics	*N*^a^ (%)
*Age*, M (***SD***)	17.32 (1.22)
***Grade***	
**SHS** 1	1,107 (46%)
**SHS 2**	573 (24%)
**SHS 3**	720 (30%)
***Ethnicity***	
**Akan**	1,860 (77%)
**Northerner**	300 (13%)
**Ewe/Guans**	121 (5%)
**Ga—Adangbe**	49 (2%)
**Other**	68 (3%)
***Program of study***	
**Science**	287 (12%)
**General arts**	1,251 (52%)
**Visual arts**	69 (3%)
**Business**	138 (6%)
**Home economics**	584 (24%)
**Agric**	59 (2%)
***Mother/female guardian education***	
**No formal education**	217 (9%)
**Primary/Junior High School**	616 (26%)
**Senior High School or higher**	1,556 (65%)
***Father/male guardian education***	
**No formal education**	171 (7%)
**Primary/Junior High School**	553 (23%)
**Senior High School or higher**	1,640 (69%)
***Ever received sexual health education by parents/guardians***	
**Yes**	1,732 (72%)
**No**	661 (28%)
***Ever received sexual health education by friends***	
**Yes**	1,492 (62%)
**No**	902 (38%)
***Ever received sexual health education by teachers***	
**Yes**	1,830 (76%)
**No**	564 (24%)
***Provision of sexual health education classes in school***	
**Yes**	834 (35%)
**No**	1,557 (65%)
***Ever received sexual health by health care provider***	
**Yes**	1,215 (51%)
**No**	1,170 (49%
***Administrative location of school***	
**District**	1,275 (53%)
**Metropolitan/municipal**	1,123 (47%)

a May not add up to 2,400 due to missing data.

### Cervical Cancer Prevention Knowledge

The overall mean knowledge score was 14.57 (median: 15, *SD*: 2.68, range: 5–23) out of a possible score of 26. No student correctly answered all items, and 68% (95% CI: [66%, 70%]) of students correctly answered more than half of all items. The percentages of correct responses for the items are presented in Table 2. Knowledge gaps reported by at least two-thirds of students included not being aware that: early sexual debut (before 18 years) was a risk factor for cervical cancer (72%); a blood test cannot detect whether you have cervical cancer (71%); and cervical cancer screening should begin in women at age 21 (69%). About half of students incorrectly believed that poor hygienic practices (47%) and activities pertaining to the introduction of foreign substances into the vagina (46%) may lead to cervical cancer, and that cervical cancer can be transmitted through blood (44%) and can be prevented by praying to God (42%).

**Table 2.** Participants’ Knowledge About Cervical Cancer Risk Factors, Primary and Secondary Prevention Strategies, and HPV-Related Knowledge. *N*=2,400.

Knowledge about cervical cancer risk factors		Correct response	
		*N* (%)	[95% CI]
Which of the following can increase chances of getting cervical cancer?			
Evidence-based items	Smoking cigarettes	921 (38%)	[37%, 40%]
	Having sexually transmitted infections e.g., HPV, HIV/AIDS	1,092 (46%)	[44%, 48%]
	Using oral contraceptives	1,059 (44%)	[42%, 46%]
	Early sexual intercourse (before 18 years)	678 (28%)	[26%, 30%]
	Having multiple sexual partners	885 (37%)	[35%, 39%]
	Having a family history of cervical cancer	835 (35%)	[33%, 37%]
Non-evidence-based items	Poor personal hygiene	1,270 (53%)	[51%, 55%]
	Blood contact of a person with cervical cancer	1,336 (56%)	[54%, 58%]
	Having one or more abortions	1,327 (55%)	[53%, 57%]
	Vaginal use of Dettol or strong soaps	1,118 (47%)	[45%, 49%]
	Spiritual forces (e.g., witchcraft)	1,929 (81%)	[79%, 82%]
	Vaginal insertion of herbs	1,294 (54%)	[52%, 56%]
Knowledge about primary prevention of cervical cancer		Correct response	
		*N* (%)	[95% CI]
Which of the following can reduce a woman’s chance of getting cervical cancer?			
Evidence-based items	Use of condom during sexual intercourse	1,343 (56%)	[54%, 58%]
	Having regular cervical check-ups	1,840 (77%)	[75%, 79%]
	Getting HPV vaccines	1,613 (67%)	[65%, 69%]
Non-evidence-based items	Praying to God	1,393 (58%)	[56%, 60%]
	Fasting to God	1,676 (70%)	[68%, 72%]
Knowledge about secondary prevention of cervical cancer		Correct response	
		*N* (%)	[95% CI]
Please indicate whether you believe each statement about cervical cancer is TRUE OR FALSE			
Evidence-based items	Cervical cancer screening can find changes to the cervix early	1,788 (75%)	[73%, 76%]
	Cervical cancer can be prevented through screening	1,547 (65%)	[63%, 66%]
	Cervical cancer can be cured when detected early.	1,791 (75%)	[73%, 76%]
	Cervical cancer screening should begin in women at age 21.	737 (31%)	[29%, 33%]
Non-evidence-based items	A blood test can detect whether or not you have cervical cancer.	705 (29%)	[28%, 31%]
HPV-related knowledge		Correct response	
		*N* (%)	[95% CI]
Please indicate whether you believe each statement about cervical cancer is TRUE OR FALSE			
Evidence-based items	HPV infection can be transmitted during sexual intercourse	1,895 (79%)	[77%, 81%]
	HPV infection can cause cervical cancer	1,837 (77%)	[75%, 78%]
	HPV infection can be prevented by a vaccine	1,628 (68%)	[66%, 70%]
Non-evidence-based items	Only girls should be vaccinated against HPV	1,395 (58%)	[56%, 60%]
	Accurate knowledge of more than half of all statements	1,633 (68%)	[66%, 70%]
	Accurate knowledge of all statements	0%	
	Overall knowledge score	M (*SD*) = 14.57 (2.68)	

HPV = human papillomavirus; HIV/AIDs = human immunodeficiency virus infection and acquired immunodeficiency syndrome.

### Correlates of Cervical Cancer Prevention Knowledge

Compared with participants in SHS Level 1, those in SHS Levels 2 and 3 had statistically significantly higher knowledge scores. Participants who received sexual health education from their parents/guardians (mean difference = 0.51 points, 95% CI: [0.18, 0.84]) and teachers (mean difference = 0.63 points, 95% CI: [0.31, 0.94]) had statistically significantly higher knowledge scores than participants who did not receive sexual health education from their parents/guardians and teachers respectively (see Table 3). Participants whose school provided sexual health education classes had statistically significant higher knowledge scores (mean difference = 0.68 points, 95% CI: [0.16, 1.20]) than those who did not have sexual health education classes in their school.

**Table 3.** Linear Mixed-Effects Regression of Overall Cervical Cancer Prevention Knowledge Score and Interactive Effects.

Variables	
Participant characteristics	Mean difference [95% CI]
Age (per year)	0.04 [−0.08, 0.16]
Grade	
SHS two	**0.85 [0.47**, 1.22]*
SHS three	**0.58 [0.21**, 0.95]*
SHS one	1
Ethnicity	
Akan	−0.17 [−0.44, 0.10]
Northerner/Ewe/Guans/Ga—Adangbe/Other	1
Program of study	
General arts/Visual arts/Business (Non-STEM)	−0.16 [−0.41, 0.09]
Science/ /Home economics/Agric (STEM)	1
Mother/female guardian education	
Primary/Junior High School	0.00 [−0.45, 0.45]
Senior High School or higher	−0.19 [−0.62, 0.24]
No formal education	1
Father/male guardian education	
Primary/Junior High School	0.32 [−0.12, 0.82]
Senior High School or higher	0.41 [−0.07, 0.89]
No formal education	1
**Family and social factors**	
Ever received sexual health education from parents/guardians	
Yes	**0.51 [0.18**, 0.84]^*^
No	1
Ever received sexual health education from friends	
Yes	0.06 [−0.19, 0.32]
No	1
**School-based system factors**	
Ever received sexual health education from teachers	
Yes	**0.63 [0.31**, 0.94]^*^
No	1
Provision of sexual health education classes in school	
Yes	**0.68 [0.16**, 1.20]^*^
No	1
Administrative location of school	
District	−0.15 [−0.47, 0.16]
Metropolitan/municipal	1
**Healthcare system factors**	
Ever received sexual health education by health care provider	
Yes	0.05 [−0.19, 0.29]
No	1
**Interactive effects**	
Provision of sexual health education classes in school × Receive sexual health education from parents/guardians	−**0.73 [**−**1.30**, −0.15]*

*Note*. ICC = intraclass correlation; STEM = science, technology, engineering and math.

Unadjusted ICC = 0.04; adjusted ICC= 0.01.

* Significant.

**Figure 1.**
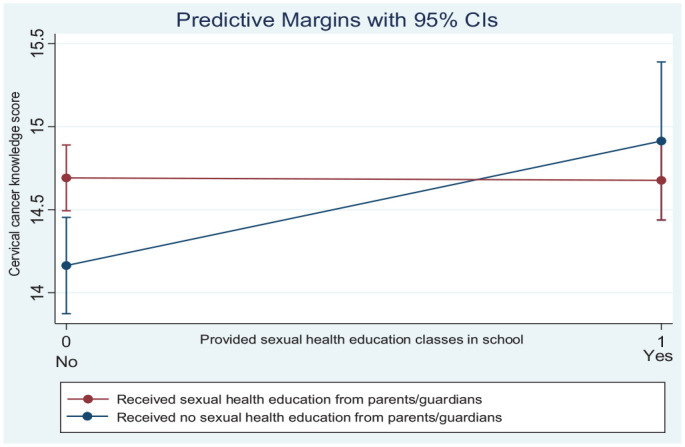
Predictive Margins of the Interactive Effects.

However, the interactive effect between receiving sexual health education from parents/guardians and the provision of sexual health education classes in schools was statistically significant (*p* < 0.05). Among students who did not receive sexual health education from their parents/guardians, those from schools that provided sexual health education classes had knowledge scores that were 0.68 points higher compared with students from schools who did not receive sexual health education classes, while the effect of school sexual health education among students who had received sexual health education from their parent/guardian was 0.73 points lower (see Table 3). This means that participants who received sexual health education from schools had higher cervical cancer knowledge scores than those who received sexual health education from parents/guardians (see Figure 1; and Table 3). A complementary interpretation of this interaction effect is that in the absence of school-based health education, parental education can increase knowledge scores. The group that received no education from school or from their parents was worse off in terms of knowledge scores (see bottom left of Figure 1). Using the Wald test, the *p* value assessed the null hypothesis that the difference in knowledge scores between those who had sexual health education at school does not differ depending on whether they had education at home. The *p* value was < 0.05, indicating a difference in knowledge scores between those who received sexual health education at school and those who received it from parents.

## Discussion

This large, cross-sectional multi-site study of female senior high school students in Ghana revealed knowledge gaps about risk factors, as well as primary and secondary prevention of cervical cancer including HPV transmission and prevention. Encouragingly, about two-thirds of students correctly responded to more than half of all knowledge items. More than half of the students correctly answered questions about cervical cancer primary prevention, and HPV infection transmission and prevention. There appeared to be more knowledge about general prevention strategies than specific cervical cancer risk factors, suggesting that students generally understand the prevention of sexually transmitted infections (STIs)-related conditions (incidentally including cervical cancer) but not the specific risk factors for this disease. This is consistent with findings for student samples from other regions of Sub-Saharan Africa (Bardaji et al., 2018; Opoku Agyemang & Manortey, 2018), Asia (Poudel & Sumi, 2019) and Europe (Bowyer et al., 2013).

Less than half of the participants had correct knowledge of the six specific cervical cancer risk factors assessed in this study: having STIs, using oral contraceptives, sexual debut before 18 years, having a family history of cervical cancer, having multiple sexual partners, and smoking cigarettes. While available evidence suggests that these modifiable lifestyle risk factors may increase the risk of cervical cancer, the risk is lower for long-term use of oral contraceptives (>5 years) than for the remaining risk factors (World Health Organization, 2014). Importantly, the effectiveness of oral contraceptives in preventing unplanned and unwanted pregnancies (with consequent prevention of burden associated with these pregnancies) far outweighs the potential risk of cervical cancer (World Health Organization, 2014). While it is important to emphasize cervical cancer risk factors during health education, the risk associated with long-term use of oral contraceptives should be communicated cautiously.

Despite the higher level of knowledge about the prevention of cervical cancer and HPV, most students did not know how cervical cancer screening was conducted or the minimum age for cervical cancer screening. The lack of understanding about the procedure and eligible age for cervical cancer screening may negatively affect screening uptake (Ampofo et al., 2020; Kirubarajan et al., 2021). These findings suggest that risk factors and prevention should be emphasized during cervical cancer education.

Misconceptions about cervical cancer risk factors and prevention strategies were prevalent. The most common of these misconceptions were that students believed activities pertaining to the introduction of foreign substances into the vagina and poor hygienic practices may lead to cervical cancer, and that cervical cancer can be transmitted through blood. These misconceptions have been previously reported by adolescents, men, and women in Africa (Mapanga et al., 2019; Moore & Driver, 2014), the United States (Morales-Campos et al., 2021), and Asia (Poudel & Sumi, 2019). While these activities are unhealthy, they are not risk factors for cervical cancer, and not unexpectedly, the latter misconceptions can lead to stigmatization of people with cervical cancer (Williams & Kenu, 2017).

A striking finding was that a large proportion of the students believed cervical cancer could be prevented by praying to God. With over 80% of the Ghanaian population being religious (including Christians and Muslims; Ghana Statistical Service, 2012), a spiritual dimension to diseases is very common. Most West Africans believe God is a supreme being who prevents diseases (Mwambazambi, 2021), making prayer and faith in God an integral part of disease prevention (Mwambazambi, 2021; Okyerefo & Fiaveh, 2017). This finding emphasizes the need to involve and partner with churches to promote the dissemination of cervical cancer messages.

Students with higher levels of education were likely to have a good understanding of cervical cancer. This can be explained by students in levels two and three having completed modules on sexual and reproductive health (SRH), substance abuse, and other general health conditions, which have been integrated into the Ghanaian senior high school curriculum (i.e., integrated science and social studies). Two main co-curricular programs are also offered as additional programs outside the regular curriculum, either during or after school: the Enhanced School Health Education Program (E-SHEP) of the Ghana Education Service; and the HIV Alert program (Awusabo-Asare et al., 2017). Given the potential benefits of these programs, their scope could be broadened (to include cervical cancer and HPV infection) and introduced early at the basic education level or right at the commencement of SHS, thus adopting a more participatory approach.

Parent and teacher communication about sexual health may play a significant role in improving cervical cancer and HPV infection knowledge. The study revealed that sexual health education provided to students by parents and teachers improved cervical cancer knowledge. Some students were able to translate this information to cervical cancer and HPV infection. This finding is consistent with previous studies that found parent-based sexual health education (Widman et al., 2019), communication about sex (Usonwu et al., 2021), and teacher-based sexual health education (Mathews et al., 2012) improved sexual health knowledge. This finding is not surprising as students spend a substantial amount of time with their teachers. Parents/guardians, on the contrary, play a critical role as socialization agents in communicating sexual health information and shaping adolescent sexual behavior. They often initiate and emphasize sexual heath discussions from a place of fear of unwanted pregnancy and STIs and the stigma of unmarried adolescents, given they have reached puberty (Usonwu et al., 2021). Female adolescents mostly believe sexual health education should be initiated by mothers at home (Shams et al., 2017) and view them as a trustworthy source of information (Awusabo-Asare et al., 2017). Collectively, these findings emphasize the need to provide parents with the skills to confidently start important but sensitive conversations about sex-related topics with their children. Sexual health training and investment in resources (including learning materials) for teachers should also be prioritized to enhance their teaching skills.

Schools and parents play significant roles in improving cervical cancer knowledge. The interaction analysis revealed that among students who have not received sexual health education from parents or guardians, sexual health education received from schools through health classes is associated with higher knowledge scores (compared with having received no sexual health education from schools). However, among students who have not received sexual health education in schools, those who received education at home had higher knowledge scores than those who did not receive education at home. Overall, these findings suggest that receiving sexual health education from either schools or parents increases cervical cancer knowledge among students, compared with not receiving any sexual health education from any source. As advocated by the World Health Organization’s HPS framework, parents should be engaged alongside incorporation and strengthening school-based cervical cancer education into the Ghanaian senior high school curriculum. School-based education improves knowledge about cervical cancer and HPV infection if there are consistent follow-up sessions (Ampofo et al., 2022). However, there is no evidence for the effectiveness of parent-based cervical cancer education, and future studies should explore this potential method. Allocating specific times for school-based SRH education will allow adequate time for students to understand and apply the information received (Awusabo-Asare et al., 2017). In addition, schools should be supported with adequate resources to provide curriculum-based SRH and extra-curricular SRH. In Ghana, schools provide extra-curricular SRH activities whereby health care providers, religious persons and peer educators commonly visit schools to teach SRH education (Awusabo-Asare et al., 2017).

### Strengths and Limitations

This is the first study to use a large multisite sample of senior high school students to comprehensively describe the extent of knowledge about cervical cancer and identify individual characteristics, the social environment (e.g., parents and peers), and broader systems (e.g., education and health) influencing knowledge. Nevertheless, there are some limitations. First, the findings of this study were restricted to public senior high school students aged 16 years and above in the Ashanti region, reducing the Generalizability of the results across other school settings in Ghana. As adequate representation of students is important for generalizing study findings, more than 65% of senior high schools are government-managed and admit the highest number of students, and the Ashanti region records the highest number of senior high schools in Ghana (Nyabor, 2017).

Second, knowledge scores may have been under/overestimated given that an author-developed study-specific survey was used because there is no standardized tool to measure cervical cancer prevention knowledge. While the reliability and validity of a measure is essential, efforts were made to develop a robust tool with minimal errors through broad consultation with participants and experts, an integrative review, and an evaluation of psychometric properties. Researchers are encouraged to validate the knowledge scale in other regions in Ghana or similar settings.

### Implications for Practice

This study’s findings provide an opportunity to broaden the scope and review the content and strategies for the teaching of sexual health education on cervical cancer in Ghanaian schools. While the content of the curricula in the core subjects is limited in scope, it appears the limited range of topics included in the integrated and social science courses are promising and could provide the basis for cervical cancer education. To reduce students’ knowledge gaps about cervical cancer, the Government of Ghana, Ministry of Education, and the E-SHEP Unit of the Ghana Education Service could introduce cervical cancer education at the primary or junior high school level, provide teachers with in-service training about cervical cancer, and equip them with appropriate resources (such as providing teaching and learning materials and more classroom time). In addition, due to the historic taboo nature of sexuality discussions, teacher communication skills about sex should be enhanced by providing a level of comfort, and empowerment to engage adolescents in interactive discussions (Usonwu et al., 2021). Alternatively, the Ghana Education Service could liaise with the Ghana Health Service to provide health educators to deliver cervical cancer education during health education classes.

## Conclusion

Female senior high school students have limited knowledge and some misconceptions about cervical cancer risk factors as well as primary and secondary prevention of cervical cancer. In particular, knowledge gaps about risk factors and secondary prevention were reported. Parents and teachers education about sexual health by parents and teachers, and school-based sexual health education increased cervical cancer knowledge. However, interactive effects indicated that sexual health education received from schools or homes alone may be adequate. Strategies to support and strengthen school- and parent-based programs to provide students with accurate cervical cancer prevention information are needed. Future research should develop and test strategies for effective implementation of cervical cancer prevention programs among female students, particularly those in the lower levels of high school.

## Supplemental Material

sj-docx-1-heb-10.1177_10901981231217978 – Supplemental material for Prevalence and Correlates of Cervical Cancer Prevention Knowledge Among High School Students in Ghana Supplemental material, sj-docx-1-heb-10.1177_10901981231217978 for Prevalence and Correlates of Cervical Cancer Prevention Knowledge Among High School Students in Ghana by Ama Gyamfua Ampofo, Lisa J Mackenzie, Shadrack Osei Asibey, Christopher Oldmeadow and Allison W Boyes in Health Education & Behavior

sj-docx-2-heb-10.1177_10901981231217978 – Supplemental material for Prevalence and Correlates of Cervical Cancer Prevention Knowledge Among High School Students in Ghana Supplemental material, sj-docx-2-heb-10.1177_10901981231217978 for Prevalence and Correlates of Cervical Cancer Prevention Knowledge Among High School Students in Ghana by Ama Gyamfua Ampofo, Lisa J Mackenzie, Shadrack Osei Asibey, Christopher Oldmeadow and Allison W Boyes in Health Education & Behavior

sj-docx-3-heb-10.1177_10901981231217978 – Supplemental material for Prevalence and Correlates of Cervical Cancer Prevention Knowledge Among High School Students in Ghana Supplemental material, sj-docx-3-heb-10.1177_10901981231217978 for Prevalence and Correlates of Cervical Cancer Prevention Knowledge Among High School Students in Ghana by Ama Gyamfua Ampofo, Lisa J Mackenzie, Shadrack Osei Asibey, Christopher Oldmeadow and Allison W Boyes in Health Education & Behavior
