# Supplementary material for: Prevalence and Correlates of Cervical Cancer Prevention Knowledge Among High School Students in Ghana
Source: Health Educ Behav. 2023 Dec 17;51(2):185–96. doi: 10.1177/10901981231217978 (PMC10981191; doi:10.1177/10901981231217978)
Supplement: sj-docx-1-heb-10.1177_10901981231217978 – Supplemental material for Prevalence and Correlates of Cervical Cancer Prevention Knowledge Among High School Students in Ghana [file sj-docx-1-heb-10.1177_10901981231217978.docx]

**Appendix A**

**Methods**

Description of study settings

The Ashanti region is the third largest of 16 administrative regions in Ghana, and has the highest population (5.4 million) (Ghana Statistical Service, 2021) and a high number of senior high schools (n = 180) (Nyabor, 2017). Of the 180 senior high schools, 127 are public and the rest are private (Nyabor, 2017). Public schools admit the highest number of students in Ghana. Senior high schools are categorised as co-educational (mixed sex) or single-sex (girls or boys only) schools (Nyabor, 2017). Additionally, the senior high schools may have three residential arrangements (boarding students only; day students only; combined boarding and day students). The senior high school (SHS) level in Ghana is equivalent to grade 9-12 in the United States of America, has three levels (SHS year one to year three), and students aged between 16 and 24 years.

Participants

*Sampling:* A multistage cluster sampling process was adopted. The first stage involved the selection of schools in the region, and the second stage involved the selection of students.

*Selection of schools*

Schools were eligible to be recruited for the study if they were mixed and single-sex (all girls) public schools. Cluster sampling was used to randomly select schools from the 122 eligible schools. Prior to selecting the 17 schools, the region was divided into northern and southern sectors using an arbitrary horizontal line to ensure adequate representation of students (Ampofo et al. 2023). Schools were further stratified by location: District (inhabitants greater than 75,000 and less than 95,000); and Municipal (inhabitants greater than 95,000 and less than 250,000)/Metropolitan (inhabitants greater than 250,000). Four sectors (strata) were identified: i) north District; ii) north Municipal/Metropolitan; iii) south District; and iv) south Municipal/Metropolitan schools.

Seventeen schools were needed to recruit 2400 subjects (with at least 141 from each school) based on the sample size calculation. Using a Microsoft Excel spreadsheet, random numbers for each school in a particular stratum were automatically generated by an independent statistician. The generated random numbers were sorted from smallest to largest in each stratum, and a total of four schools (i.e., four random numbers) were selected from each of the four strata (n=16 schools). For the final seventeenth school to be selected, one stratum was randomly selected from the four strata, and the fifth school (i.e., fifth random number) was selected from the existing sorted list for that stratum. The selected schools (n=17) consisted of 2 single-sex girls’ schools and 15 co-educational schools.

*Selection of students*

Using quota sampling, students were recruited from all three SHS levels. As cervical cancer directly affects females, eligible students were females aged 16 years and older who could provide parent/guardian consent (for those aged 16-17 years).

Procedure

*Recruitment of schools*

Permission to conduct the study was obtained from the District Directorates of the eligible schools. Following the selection of schools, letters were sent to the District Directors of Education informing them about the survey and requesting their support. For directorates that responded favourably to the request, the research team sent individual letters about the study to the selected schools including a cover letter, study information and written consent form.

Heads of all sampled schools were visited by a research team member to solicit their support for and participation in the study. If necessary, follow-up contact was made by phone. Some heads of school solicited approval from the schools’ boards. Signed consent from each school’s head was obtained before commencement of student recruitment. The first four schools from the randomly generated list in each stratum were approached by a member of the research team and invited to participate. It was intended that if a school declined to participate, the next school on the list in the stratum would be approached until the required number of schools was achieved. However, as the first four schools in each stratum agreed to participate, this intended step was not required.

*Recruitment of students*

Once approval was granted by each head of school, students were verbally informed by the research team about the study during the school’s general assembly. Eligible students were invited to visit the school’s designated classroom for the study during their break periods. Following Public Health orders in response to the COVID-19 pandemic, tables and chairs in the designated classroom were arranged 1.5m apart, and masks, sanitiser and pens were provided to students to maintain their health and safety. Students were informed verbally and in writing that a) their decision to participate was completely voluntary, b) that their responses were anonymous, c) that they could withdraw from the study at any time, and d) completion of the survey would be taken as an indication of their voluntary informed consent to participate. This consent statement, which met international standards for consent and was approved by the appropriate human ethics board, was provided in writing on the cover sheet of the survey.

Students who came to the designated classroom completed an anonymous pen-and-paper survey assessing sociodemographic characteristics and knowledge about cervical cancer. To ensure students' confidentiality and privacy, they were asked to put the completed survey in a sealed envelope and place it in allocated boxes at the front of the room. All information provided by respondents was treated confidentially and was accessible only by the research team. To maintain students’ anonymity, neither heads of schools nor teachers knew which students completed the survey. For students under 18 years, written parental/guardian consent was obtained before surveys were provided and completed. Recruitment continued until the targeted number of students (at least 141) was achieved for each school.

**References**

Ampofo, A. G., Boyes, A. W., Asibey, S. O., Oldmeadow, C., & Mackenzie, L. J. (2023). Prevalence and correlates of modifiable risk factors for cervical cancer and HPV infection among senior high school students in Ghana: a latent class analysis. BMC Public Health, 23(1), 340. doi: 10.1186/s12889-022-14908-w

Ghana Statistical Service. (2021). 2021 Population and housing census report (Vol. 3A). Ghana: Ghana Statistical Service.

Jonas Nyabor. (2017). Regional distribution of Senior High Schools in Ghana. Retrieved 8th January 2022, from <https://citifmonline.com/2017/09/regional-distribution-of-senior-high-schools-in-ghana-infographic/>
