# Supplementary material for: Prevalence and Correlates of Cervical Cancer Prevention Knowledge Among High School Students in Ghana
Source: Health Educ Behav. 2023 Dec 17;51(2):185–96. doi: 10.1177/10901981231217978 (PMC10981191; doi:10.1177/10901981231217978)
Supplement: sj-docx-2-heb-10.1177_10901981231217978 – Supplemental material for Prevalence and Correlates of Cervical Cancer Prevention Knowledge Among High School Students in Ghana [file sj-docx-2-heb-10.1177_10901981231217978.docx]

**Interview guide for pilot study**

**V2 dated 01122020**

**Introduction**

**Sample questions/prompts**

1. **What do you understand by term?**

- Cervical cancer
- Cervical cancer screening

1. What causes cervical cancer?
2. How can cervical cancer be prevented?
3. How you feel about cervical cancer?
4. How serious is cervical cancer to you?
5. **Tell me more about how you feel about cervical cancer screening**

***Prompts***

1. What do you like/dislike about cervical cancer screening?
2. What do you see are the advantages of having cervical cancer screening?
3. What do you see are the disadvantages of having cervical cancer screening?
4. **Can you tell me more about who or what will influence your decision to get cervical cancer screening?**

***Prompts***

1. Who would support to get screened for cervical cancer?
2. Who would be against your decision to have cervical cancer screening?
3. Whose opinion is important to you when deciding to have cervical cancer screening?
4. Whose action will influence your decision to have cervical cancer screening?
5. **Can you tell me more about what makes it difficult or easier to get cervical cancer screening?**

***Prompts***

1. What things make it easy for you to have cervical cancer screening?
2. What things make it hard for you to have cervical cancer screening?
3. **Can you tell me more about the things that could get in your way if you wanted to have cervical cancer screening?**

**Prompts**

1. What kinds of things would help you overcome any barriers to have cervical cancer screening?
2. **What do you understand by term?**

- Human papillomavirus (HPV)
- Vaccine (injection)

1. What causes HPV?
2. How can HPV be prevented?
3. How you feel about HPV?
4. How serious is HPV to you?
5. **Tell me more about how you feel about cervical cancer injections**

***Prompts***

1. What do you like/dislike about injection?
2. What do you see are the advantages of having injection?
3. What do you see are the disadvantages of having injection?
4. **Can you tell me more about who or what will influence your decision to get injection?**

***Prompts***

1. Who would support to get injection?
2. Who would be against your decision to have injection?
3. Whose opinion is important to you when deciding to have injection?
4. Whose action will influence your decision to have injection?
5. **Can you tell me more about what makes it difficult or easier to get an injection?**

***Prompts***

1. What things make it easy for you to have injection?
2. What things make it hard for you to have injection?
3. **Can you tell me more about the things that could get in your way if you wanted to have an injection?**

***Prompts***

1. What kinds of things would help you overcome any barriers to have injection?
2. **Can you tell me more about where, how and who you would like to receive cervical cancer information from?**
3. Is it a health professional? at your school or church e.t.c?
4. Would you like the information to be delivered through face-to-face, online, e.t.c?
