# Supplementary material for: Prevalence and Correlates of Cervical Cancer Prevention Knowledge Among High School Students in Ghana
Source: Health Educ Behav. 2023 Dec 17;51(2):185–96. doi: 10.1177/10901981231217978 (PMC10981191; doi:10.1177/10901981231217978)
Supplement: sj-docx-3-heb-10.1177_10901981231217978 – Supplemental material for Prevalence and Correlates of Cervical Cancer Prevention Knowledge Among High School Students in Ghana [file sj-docx-3-heb-10.1177_10901981231217978.docx]

**Cervical Cancer Survey**

Version 3, dated 18032021

**SECTION A: Knowledge of cervical cancer**

*The following questions are about your knowledge of things or activities that can lead to cervical cancer. For each question, please tick the box (🗹) which best describes your answer. If you are unsure about a question, please give the best answer that you can.*

| 1. Which of the following can increase chances of getting cervical cancer*? Cervical cancer is a disease that can affect the tip of the womb.* | | Yes | | No |  |
| --- | --- | --- | --- | --- | --- |
| 1. Smoking cigarettes | | _1_🞎 | | _2_🞎 |  |
| 1. Having sexually transmitted infections e.g., Human Papillomavirus, HIV/AIDS | | _1_🞎 | | _2_🞎 |  |
| 1. Using oral contraceptives | | _1_🞎 | | _2_🞎 |  |
| 1. Having one or more abortions | | _1_🞎 | | _2_🞎 |  |
| 1. Having sex before 18 years of age | | _1_🞎 | | _2_🞎 |  |
| 1. Applying Dettol or strong soaps into the vagina during bathing | | _1_🞎 | | _2_🞎 |  |
| 1. Having had more than one sexual partner over a lifetime | | _1_🞎 | | _2_🞎 |  |
| 1. Spiritual forces (e.g. witchcraft) | | _1_🞎 | | _2_🞎 |  |
| 1. Inserting herbs into the vagina | | _1_🞎 | | _2_🞎 |  |
| 1. Having a family history of cervical cancer | | _1_🞎 | | _2_🞎 |  |
| 1. Poor personal hygiene | | _1_🞎 | | _2_🞎 |  |
| 1. Contact with blood of a person with cervical cancer | | _1_🞎 | | _2_🞎 |  |
|  | |  | |  |  |
| 1. Which of the following can reduce a woman’s chance of getting cervical cancer? | | **True** | | **False** |  |
| 1. Praying to God | | _1_🞎 | | _2_🞎 |  |
| 1. Fasting to God | | _1_🞎 | | _2_🞎 |  |
| 1. Using a condom during sexual intercourse | | _1_🞎 | | _2_🞎 |  |
| 1. Having regular cervical check ups | | _1_🞎 | | _2_🞎 |  |
| 1. Getting vaccinated with HPV vaccines to prevent cervical cancer   (*A vaccine is a liquid often injected into the arm)* | | _1_🞎 | | _2_🞎 |  |
| 1. Please indicate whether you believe each statement about cervical cancer is TRUE OR FALSE. | | **True** | | **False** |  |
| 1. A blood test can detect whether or not you have cervical cancer. | | _1_🞎 | | _2_🞎 |  |
| 1. Cervical cancer screening/check-up can find changes to the cervix early. | | _1_🞎 | | _2_🞎 |  |
| 1. Cervical cancer screening/check-up should begin in women at age 21. | | _1_🞎 | | _2_🞎 |  |
| 1. Cervical cancer can be prevented through screening/check-up. | | _1_🞎 | | _2_🞎 |  |
| 1. Cervical cancer can be cured when detected early. | | _1_🞎 | | _2_🞎 |  |
| 1. Please indicate whether you believe each statement about HPV is TRUE OR FALSE. *(HPV is an infection).* | **True** | | **False** | | |
| 1. HPV infection can be passed on from one person to another during sexual intercourse | _1_🞎 | | _2_🞎 | | |
| 1. HPV infection can cause cervical cancer | _1_🞎 | | _2_🞎 | | |
| 1. HPV infection can be prevented by a vaccine | _1_🞎 | | _2_🞎 | | |
| 1. Only girls should be vaccinated against HPV | _1_🞎 | | _2_🞎 | | |

**Thank you for completing this survey. Your time is greatly appreciated.**

**If you have any comments, please write them in the space below.**

………………………………………………………………………………………………………………………………………………………………………………………………………………………………………………………………………………………………………………………………………………………………………………………………………………………………………………………………
